# The P2X7 Receptor as Regulator of T Cell Development and Function

**DOI:** 10.3389/fimmu.2020.01179

**Published:** 2020-06-10

**Authors:** Fabio Grassi

**Affiliations:** Faculty of Biomedical Sciences, Institute for Research in Biomedicine, Università della Svizzera Italiana, Bellinzona, Switzerland

**Keywords:** P2X7, T cell, extracellular ATP (eATP), T cell effector function, mucosal immunology, T cell development

## Abstract

Unique structural features characterize the P2X7 receptor with respect to other P2X family members. Dual gating by eATP and regulated expression of P2X7 can imprint distinct outcomes to the T cell depending on the metabolic fitness and/or developmental stage. In the thymus, signaling by P2X7 contributes to γδ T cell lineage choice. In secondary lymphoid organs, P2X7 stimulation promotes Th1/Th17 polarization of CD4^+^ naïve cells, Tregs conversion to Th17 cells and cell death of Tfh cells that are not stimulated by cognate antigen. Moreover, P2X7 stimulation in eATP rich microenvironments, such as damaged and/or inflamed tissues as well as tumors, induces cell death of various T cell effector subsets.

## Introduction

Signaling by adenosine triphosphate (ATP) emerged very early in evolution and is involved in the regulation of highly diverse biologic functions. Trimeric ATP-gated ionotropic P2X receptors are amongst the most ancient signaling channels, having been present in single-cell protozoa and algae (1). The first evidence of T cell responsiveness to extracellular ATP (eATP) dates back to 1989, when Di Virgilio et al. showed that eATP induced plasma membrane depolarization and permeability to low MW dyes, possibly leading to cell death (2). It was then hypothesized that endogenously generated eATP promoted the effector function of cytotoxic T cells via purinergic receptors (3). Subsequent experiments indicated that activation of P2X receptors in T cells could contribute to the outcome of TCR stimulation both in murine and human cells (4, 5). As in other cells of the immune system, the P2X7 receptor subtype stands out among P2X family members as the most important regulator of T cell function. It is a non-selective cationic channel characterized by dual gating: receptor exposure to low concentrations of ATP (e.g., micromolar range) results in small-amplitude currents, whereas stimulation with ATP in the hundreds micromolar range leads to opening of a cytolytic pore and cell death (6). Cryoelectron microscopy of the rat receptor in apo (closed pore) and ATP-bound (open pore) states has unraveled structural insights into P2X7 architecture, which confer the functional peculiarities that distinguish it from the other P2X family members, namely low affinity for ATP, lack of desensitization and cell death initiation (7). In particular, P2X7 combines a P2X domain with a unique “C-cysteine anchor” intra-cytoplasmic motif and a C-terminal cytoplasmic ballast domain (which contains a Zn coordinating cysteine motif and a GDP-binding region), both of which are not present in other P2X receptors. The C-terminal region of P2X7 has been recently hypothesized to originate from the capture of a ballast domain by a P2X gene in ancestral jawed vertebrates (8).

## Signaling by P2X7

The human and mouse genes encoding for P2X7 are located in syntenic regions of chromosome 12 and 5, respectively, in close proximity with the gene encoding the P2X4 receptor. Numerous splice variants have been identified for the P2X7 receptor in different species, however, the functional characterization of the various protein isoforms is largely incomplete [reviewed in (9)]. The *P2RX7* gene is highly polymorphic and single nucleotide polymorphisms (SNPs) can significantly influence the functional properties of the receptor (10). Genetic association studies support non-synonymous SNPs (NS-SNPs) in the *P2RX7* gene as an important genetic factor that alters the susceptibility of individuals to various pathological conditions. The predominant expression of P2X7 in cells of the immune system correlates with detection of NS-SNPs in diseases, in which immune system cells play a pivotal role in the pathogenesis [reviewed in (11)].

In addition to eATP, non-nucleotide agonists, including cathelicidins, amyloidogenic peptide β, and serum amyloid, have been suggested to activate P2X7 or act as positive allosteric effectors (10). Moreover, the murine P2X7 receptor can be ADP-ribosylated by the ADP-ribosyltransferase 2.2 (ART2.2) that catalyzes the transfer of ribose from nicotinamide adenine dinucleotide (NAD^+^) to R125 in the ectodomain of the P2X7 receptor, resulting in its activation (12). In T cells, P2X7 activation by ADP-ribosylation causes calcium flux, phosphatidylserine exposure, shedding of L-selectin (CD62L), cell shrinkage, pore formation and propidium iodide uptake (13). This alternate mechanism of P2X7 activation is not observed in humans, which lack ART2.1 and ART2.2 (14), and is particularly relevant in murine T cells compared to other cells because of the specific expression of a P2X7 splice variant, that is sensitive to activation by ADP-ribosylation (15–17). The high sensitivity of immunosuppressive T regulatory cells (Tregs) to depletion by NAD^+^ released during cell damage or inflammation led to hypothesize a function for the ART2-P2X7 pathway in murine Tregs homeostasis (18). An important consequence of P2X7 gating by ADP-ribosylation is the “spontaneous” P2X7 activation of T cells (19) and reduced vitality of Tregs, tissue-resident memory (Trm) (20) and natural killer T cells (21) that co-express high levels of ART2.2 and P2X7, during the isolation procedure from mice. This phenomenon has been successfully counteracted by the injection of ART2.2-blocking nanobodies prior to organ harvesting (20, 22). The shedding of CD62L mentioned above as well as of CD27 and IL-6 receptor (IL-6R) by P2X7 stimulation, are due to P2X7-mediated activation of metalloproteases, such as ADAM10 and ADAM17 (23–25). Since CD62L promotes T cell homing to secondary lymphoid organs (SLOs), P2X7 activation in naïve T cells stimulated by cognate antigen might promote their egress from SLOs. Interestingly, Tregs expressing the ATP-degrading enzyme ectonucleoside triphosphate diphosphohydrolase-1 (CD39) ameliorated contact hypersensitivity reactions by suppressing ATP-induced CD62L shedding and promoting CD8^+^ cells retention in skin-draining lymph nodes (LNs) (26). Another possible important target of P2X7 induced metalloprotease activation in T cells is CD27, a member of the tumor necrosis factor receptor family, which supports antigen-specific expansion and T cell memory generation (27, 28). Since CD27 activation by interaction with its ligand CD70 is crucial for the outcome of T cell response (29), P2X7-mediated shedding of CD27 might contribute to the regulation of adaptive immunity and/or immunopathology. Along another line, the induction of IL-6R shedding by P2X7 could condition T cell polarization toward pro-inflammatory vs. immunosuppressive programs. These observations indicate the pleiotropic role this P2X7 feature might have in conditioning T cell function.

## P2X7 in T Cell Development

**Figure 1:**
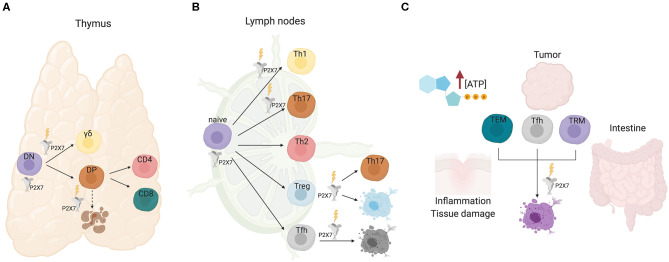
P2X7 activity in T cell development and function. **(A)** In the thymus, P2X7 activity could promote differentiation of immature DN cells. Signaling by P2X7 contributes to γδ lineage choice by promoting ERK phosphorylation and induction of early growth response (*Egr*) transcripts. Conversely, progression to the DP and single positive CD4 or CD8 stages is not influenced by P2X7, albeit P2X7 might function in cell death induction of neglected DP cells. **(B)** In secondary lymphoid organs, P2X7 stimulation promotes Th1/Th17 polarization of CD4^+^ naïve cells, Tregs conversion to Th17 cells and cell death of Tfh cells that are not stimulated by cognate antigen. **(C)** Sensitivity of TEM, Tfh, and tissue resident memory T cells to P2X7 mediated cell death in eATP rich microenvironments.

αβ and γδ T cell development in the thymus is characterized by transition of thymocytes through multiple checkpoints, most of which are regulated by the rearrangement status and specificity of the clonotypic TCR. Whereas, γδ cells develop from CD4^−^8^−^ double negative (DN) thymocytes, αβ cells progress from DN to mature MHCI and MHCII restricted CD8^+^ and CD4^+^ T cells, respectively, through an intermediate CD4^+^8^+^ double positive (DP) stage, in which TCR specificity dictates either positive or negative selection of cells (30). The analysis of the dynamics of changes in cytosolic Ca^2+^ elicited by eATP in thymocytes via P2X7 receptor showed significant variations between individual cells that were dependent on the developmental stage. It was hypothesized that eATP could promote differentiation of most immature DN cells in the outer cortex; conversely, progression to the DP stage in the inner cortex would correspond to loss of responsiveness to eATP via P2X7, thus protecting positively selected cells from eATP released during massive apoptosis of neglected or negatively selected DP cells (31). More recently, this phenomenon was explained by the demonstration of the direct binding of histone deacetylase (HDAC) 3 to the *P2rx7* enhancer and repression of P2X7 signaling in DP cells (32). Nevertheless, protection of DP cells from death by pharmacological P2X antagonism could suggest some function of P2X7 in the elimination of neglected DP cells [(33); Figure 1A].

TCR signal strength is a crucial determinant in T cell fate. Increased signal strength of γδTCR with respect to pre-TCR results in induction of the γδ differentiation program. P2X7 signaling contributes to γδ lineage choice by promoting ERK phosphorylation and induction of early growth response (*Egr*) transcripts. Moreover, the impairment of the ERK-Egr-inhibitor of differentiation 3 (Id3) signaling pathway in γδ cells from *P2rx7*^−/−^ mice resulted in diversion of γδ T cells to “innate-like” NK1.1-expressing cells with limited TCR diversity (34). These experiments suggest a function of P2X7 in shaping the γδ T cells repertoire, whereas lineage choice and differentiation to mature CD4^+^ or CD8^+^ αβ thymocytes do not seem to be affected by P2X7 expression (Figure 1A). Whether and how P2X7 activity might influence cell metabolism in conditioning γδ thymocytes differentiation has not been addressed so far.

## P2X7 in naïve T Cell Response

In T cells, the increase in the concentration of cytosolic Ca^2+^ that follows TCR stimulation by peptide/MHC complex is accompanied by mitochondrial uptake of Ca^2+^. This phenomenon avoids cellular Ca^2+^ overload, and contribute to a rapid clearing of Ca^2+^ in spatially restricted areas, such as near Ca^2+^ channels in the plasma membrane or the ER (35). Moreover, mitochondrial uptake of Ca^2+^ stimulates the aerobic synthesis of ATP (36, 37). TCR triggering of naïve T cells results in ATP release via pannexin-1 hemichannels and autocrine stimulation of P2X receptors in the plasma membrane. Murine naïve CD4 T cells express *P2rx1, P2rx4* transcripts, and higher levels of *P2rx7*. The ATP released upon naïve T cell activation functions as an autocrine stimulus and sustains MAPK signaling and induction of pro-inflammatory programs via P2X receptors stimulation (Figure 1B). Accordingly, pharmacological antagonism of P2X activity promoted T cell anergy and showed beneficial effects in autoimmune conditions (38). These effects were also favored by the conversion of naïve CD4 T cells into immunosuppressive T regulatory cells (Tregs) (39). Autocrine signaling by eATP via P2X7 receptor was shown to contribute to TCR-mediated Ca^2+^ influx, NFAT activation and IL-2 production in human CD4 T cells; blocking of P2X7 signaling inhibited T cell activation, suggesting P2X7 receptor is required for effective T cell activation (40). Importantly, expression of CD39 and CD73, the ecto-5′-nucleotidase that degrades extracellular AMP into adenosine, by other immune and tissue resident cells can dramatically condition the outcome of T cell responses (41–43). The *P2xr7* gene is robustly upregulated in T effector/memory (TEM) cells. P2X7 activity seems to play different functions in regulating the proliferative response of naïve vs. TEM cells upon TCR stimulation. Murine *P2rx7*^−/−^ CD4 naïve cells did not show any difference in cell proliferation as compared to WT cells upon TCR stimulation, suggesting that P2X1 and/or P2X4 could compensate for the lack of P2X7 activity, an observation made also in human T cells (44). In contrast, stimulation of *P2rx7*^−/−^ TEM cells revealed a peculiar enhancement of cell cycling activity with respect to the WT counterpart (our unpublished observations). This phenomenon could be due to the sustained generation of mitochondrial reactive oxygen species (ROS) that was associated to P2X7 activity in T cells (45), and induction of premature cellular senescence.

## P2X7 Activity in Effector/Memory T Cell Function

Extracellular ATP is virtually absent in the interstitium of tissues in physiological conditions with the notable exception of the intestine, where eATP generated by the microbiota can permeate enterocytes (46). In contrast, damaged and/or inflamed tissues as well as tumors' microenvironment (TME) are characterized by eATP concentrations that can reach the millimolar range (47–49). Therefore, P2X7 expression can crucially impact the outcome of local immune system response. In this respect, we have shown that P2X7 stimulation in immunosuppressive T regulatory cells (Tregs) can result in conversion into pro-inflammatory IL-17 secreting cells, thereby possibly worsening the inflammatory tissue damage in pathological conditions [(39); Figure 1B]. Analogously, P2X7 receptor inhibition promoted long-term cardiac transplant survival in murine recipients of fully mismatched allograft by reducing T cell activation and Th1/Th17 differentiation (50).

In T follicular helper (Tfh) cells, conversely, P2X7 stimulation restricts the expansion of aberrant cells and the generation of self-reactive antibodies in experimental murine lupus, but its activity is dispensable for regulation of antigen-specific Tfh cells during parenteral vaccination. P2X7 stimulation likely controls the development of pathogenic ICOS^+^ IFN-γ-secreting Tfh cells, which characterize systemic lupus erythematosus (SLE), by inducing pyroptosis via caspase-mediated activation of gasdermin D (Figure 1B). Notably, SLE patients are characterized by reduced P2X7 activity in circulating Tfh cells (51). Acute TCR stimulation of Tfh cells robustly downregulates *P2rx7* expression, thus protecting antigen responding T cell from cell death (52). Similar results have been obtained in tissue resident memory T cells, suggesting that selective downregulation of *P2rx7* in T cells that productively respond to cognate antigen would ensure the amplification of pathogen-destructing cells during infections (53). In contrast, P2X7 activity is required for the establishment and maintenance of long-lived central and tissue-resident memory CD8 T cells in mice, probably reflecting the function of P2X7 as ion channel in promoting mitochondrial function and metabolic fitness (54).

## P2X7-Mediated T Cell Conditioning in the Intestine

The intestinal microbiota influences host physiology, metabolism, and immune system homeostasis. The interaction between microbes and mammalian immune system results in the selection and “tolerance” of beneficial species. Within this inter-kingdom relationship, eATP plays an important role as a released bacterial metabolite capable of modulating immune system function. The first evidence that commensal bacteria-derived ATP could condition host immune system was provided by Atarashi et al. by showing that a CD70^high^CD11c^low^ subset of lamina propria cells could be activated by intestinal ATP and induce the differentiation of pro-inflammatory Th17 cells (55). Extracellular ATP was shown to activate dendritic cells (DCs) via P2X7, thereby polarizing the T cell response in a number of physiological and pathophysiological conditions (48, 49, 56–60). However, whether P2X7 stimulation in DCs was responsible for the induction of Th17 cells by intestinal microbiota-derived eATP was not established. Signaling by P2X7 is responsible for cell death of Tfh cells in the Peyer's patches of the small intestine by bacteria-derived ATP, a mechanism important in ensuring controlled generation of T cell dependent secretory IgA (52) and a beneficial shaping of gut microbiota composition (61). The intestinal microenvironment profoundly influences the sensitivity of intraepithelial CD8 cells, both the CD8αβ and CD8αα expressing subset, to P2X7 mediated cell death. In fact, retinoic acid causes up-regulation of P2X7 on purified CD8 T cells and induces responsiveness to extracellular nucleotides. Accordingly, lack of P2X7 led to enhanced CD8^+^ T cell responses in the intestinal mucosa, thus defining P2X7 as a regulatory element in the control of CD8^+^ T cells in the intestinal mucosa (62). The induction of P2X7 upregulation by retinoic acid was observed also in CD4^+^ effector T cells. Hashimoto-Hill et al. showed retinoic acid receptor α binding to an intragenic enhancer region of the *P2rx7* gene (63). Probably, this transcriptional control is responsible for the robust expression of P2X7 on most intestinal αβ and γδ T cells, including T-helper type 1 (Th1) and Th17 cells as well as invariant NKT cells (64). Intestinal effector T cells are effectively deleted by P2X7 mediated cell death and P2X7 activation suppressed T-cell-induced colitis in lymphopenic mice (Figure 1C). Results obtained with vitamin A-deficient and *P2rx7*^−/−^ mice indicate that the retinoic acid-P2X7 pathway is important in preventing expansion of aberrantly activated T cells, as observed with “P2X7-hypoactive” Tfh cells in SLE (51). Therefore, it appears that retinoic acid controls intestinal effector T-cell populations by inducing P2X7 expression. This pathway is likely responsible also for P2X7 mediated control of Tfh cells response to oral vaccination, thereby limiting the generation of high-affinity secretory IgA (46).

## Concluding Remarks

Dual gating and regulated expression of P2X7 can imprint distinct outcomes to the T cell depending on the metabolic fitness and/or developmental stage via autocrine signaling or microenvironment's clues, like eATP or other factors (e.g., NAD^+^ in mice) conditioning P2X7 activity. The peculiarity of P2X7 function as cationic channel and cytolytic pore could be responsible for some apparently contradictory findings on P2X7 dependent responses in particular T cell subsets in different experimental settings. It would be important to define molecular mechanisms that could affect P2X7 activity in T cells (e.g., gene polymorphism, RNA splicing, microRNAs, long non-coding RNAs) in different physiological and pathophysiological contexts.

## Data Availability Statement

The original contributions presented in the study are included in the article/supplementary materials, further inquiries can be directed to the corresponding author/s.

## Author Contributions

The author confirms being the sole contributor of this work and has approved it for publication.

## Conflict of Interest

The author declares that the research was conducted in the absence of any commercial or financial relationships that could be construed as a potential conflict of interest.
